# Extensive hyperpigmented psoriatic plaques on the bilateral lower extremities

**DOI:** 10.11604/pamj.2025.52.154.49818

**Published:** 2025-12-10

**Authors:** Pawan Banduji Itankar, Gaurav Rajendra Sawarkar

**Affiliations:** 1 Department of Rachana Sharir, Mahatma Gandhi Ayurved College Hospital and Research Centre, Datta Meghe Institute of Higher Education and Research (Deemed to be University), Sawangi (Meghe), Wardha, Maharashtra, India

**Keywords:** Psoriasis, arthritis, antihistamines

## Image in medicine

A 52-year-old male manual labourer from a humid, tropical region presented with persistent reddish-black, scaly lesions on both lower limbs, extending from the knees to the ankles, for the past four months. The lesions began as mild dryness and scaling but progressively worsened, especially with scratching. He reported frequent itching, particularly at night, occasional pain, and a foul odour from the lesions, suggesting possible secondary bacterial infection. There was no history of systemic illness, trauma, or new medications. Over-the-counter emollients and antihistamines provided only temporary relief. On examination, well-demarcated maculopapular plaques with scaling, raised edges, and hyperpigmentation were noted on both lower limbs. Multiple joint pain, swelling, and stiffness were observed. Based on the clinical findings, a diagnosis of psoriatic arthritis (PA) was considered, as psoriasis commonly precedes joint involvement. Psoriasis is a chronic inflammatory skin condition characterized by scaly, erythematous plaques, and psoriatic arthritis is a frequent complication that affects the joints. The foul odour and rough skin texture suggest secondary infection from chronic scratching. Treatment includes topical corticosteroids (e.g., clobetasol) to reduce inflammation, vitamin D analogues (e.g., calcipotriene) to control scaling, phototherapy if needed, systemic agents (methotrexate or biologics) if joint symptoms arise, and antibiotics for secondary infection. Prognosis involves a chronic, relapsing course requiring long-term management and regular follow-up to prevent joint damage.

**Figure 1:**
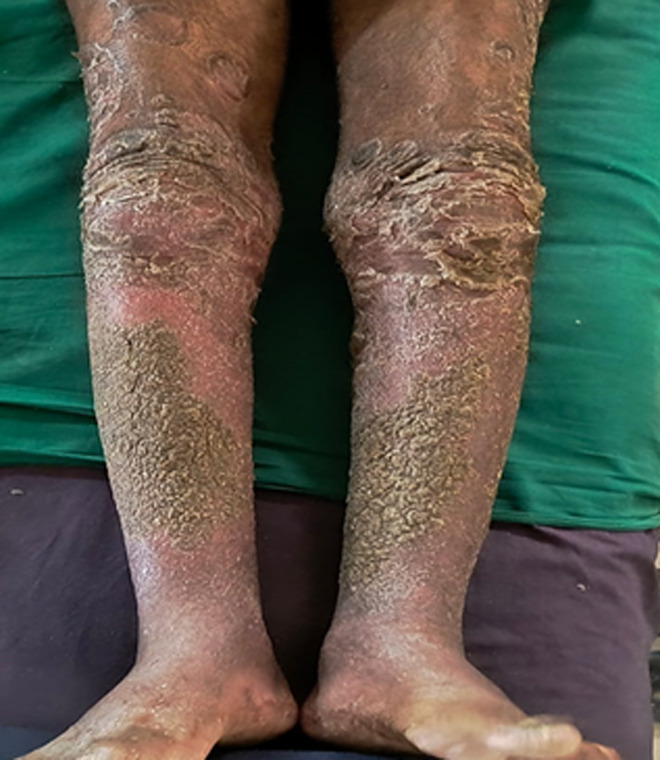
maculopapular plaques with scaling, raised edges, and hyperpigmentation on both lower limbs from the knee to the ankle

